# Analytical study on intraoperative temperature management for reducing complications in cardiac surgery

**DOI:** 10.6026/973206300213076

**Published:** 2025-09-30

**Authors:** Hiren Aswani, Sailesh I.S Kumar, Satya Sudhakara Bhat, Harish Sidharth Veerachamy, Omar Imtiaz Usmani

**Affiliations:** 1 Department of Physiology, K.J Somaiya Medical College and Research Centre, Mumbai, Maharashtra, India; 2 Department of Surgery, Madras Medical College, Tamil Nadu, India; 3 Department of Trauma and Orthopaedics, Lancashire Teaching Hospital NHS Foundation Trust, United Kingdom; 4 Department of General Medicine, Tirunelveli Medical College, Tamil Nadu, India; 5 Department of General Medicines, Era's Lucknow Medical college, Lucknow, Uttar Pradesh, India

**Keywords:** Heart surgery, postoperative results, surgical problems, hypothermia, normothermia, intraoperative temperature

## Abstract

Intraoperative temperature control affects postoperative problems following heart surgery. Hence, a group of 128 adult patients who were having elective heart surgery were observed. According to normothermic and hypothermic temperature control techniques, patients were divided into groups. The normothermic group experienced noticeably fewer problems, such as bleeding and arrhythmias, according to the results. Thus, we show critical accurate heat management is to better surgical outcomes.

## Background:

An essential part of anaesthetic and surgical treatment is intraoperative temperature control, especially in cardiac surgery where problems are a known possibility [1]. Maintaining a constant core body temperature of 37°C is essential for survival, through maintaining optimal organ, tissue and cellular function. Tight temperature regulation is achieved through a complex, integrated system of thermogenesis and heat loss. Different levels of hypothermia for defined periods of time can be protective. Temperature management during and after cardiac surgery, as well as after accidental hypothermia can have major effects on both immediate and long-term outcome [2, 3]. Historically, hypothermia has been utilized to lower metabolic demand during cardiopulmonary bypass, protecting important organs [4]. But according to new research, unchecked or excessive cooling may raise the risk of negative consequences like coagulopathy, infections, extended hospital stays and arrhythmias [5]. On the other hand, preserving normothermia has been linked to greater overall recovery, decreased inflammatory responses and enhanced coagulation profiles [6]. There is still disagreement on the ideal intraoperative temperature range, despite increased interest in thermal regulation techniques. Therefore, it is of interest to analyse the connection between intraoperative temperature control and the prevalence of postoperative problems in patients having heart surgery.

## Materials and Methods:

This 12-month analytical cohort research involved 128 adult patients at a tertiary care facility undergoing elective heart surgery (valve replacements and coronary artery bypass grafting). Group A (normothermia: 36-37°C) and Group B (moderate hypothermia: 32-35°C) were the two groups of patients that were separated according to the intraoperative temperature management method. Continuous temperature monitoring was done with oesophageal probes and forced-air warming devices and cardiopulmonary bypass settings were used to maintain thermal control. Patient demographics, comorbidities, length of surgery, blood loss, arrhythmias, wound infections and intensive care unit stay were all recorded. Exclusion criteria included emergency surgeries, pre-existing coagulopathies and intraoperative conversion to off-pump techniques. Outcomes were compared between groups using descriptive statistics and appropriate analytical tests.

## Results:

Patients managed with normothermia experienced significantly fewer postoperative complications compared to those managed under mild hypothermia. Differences were notable in outcomes such as arrhythmias, bleeding, ICU stay and infection rates, suggesting that tighter thermal regulation positively influenced recovery. Table 1 (see PDF) shows patients in both groups had comparable baseline demographics, ensuring uniformity for outcome comparisons. Table 2 (see PDF) shows the types of surgeries performed were similar across both groups, with no significant difference in distribution. Table 3 (see PDF) shows operative duration was slightly shorter in the normothermic group, but not statistically significant. Table 4 (see PDF) shows postoperative arrhythmias were significantly less common in the normothermic group. Table 5 (see PDF) shows mean blood loss and transfusion requirements were significantly lower in the normothermic group. Table 6 (see PDF) shows postoperative infections were more frequent in the hypothermic group. Table 7 (see PDF) shows ICU and hospital stay durations were notably shorter in the normothermic group. Table 8 (see PDF) shows ventilator dependency was reduced in the normothermic cohort. Table 9 (see PDF) shows postoperative lactate levels, a marker of perfusion, were lower in the normothermic group. Table 10 (see PDF) shows reoperation for bleeding was significantly higher in the hypothermic group.

## Discussion:

In order to improve postoperative outcomes following heart surgery, intraoperative temperature control is crucial, according to the analytical study's findings [7]. The incidence of arrhythmias, blood loss, infections and intensive care unit stays was much lower in patients treated with normothermia than in those treated with mild hypothermia [8]. According to new research, hypothermia protects the heart during cardiopulmonary bypass, but it can also affect coagulation and immunological function, which can lead to higher morbidity [9]. The normothermic group's lower lactate levels and shorter mechanical breathing duration further suggest improved systemic perfusion and recuperation [10]. In order to reduce problems and maximize recovery for patients undergoing heart surgery, these findings corroborate the increasing clinical preference for maintaining intraoperative temperatures close to physiological levels [11].

## Conclusion:

When compared to mild hypothermia, intraoperative normothermia dramatically lowers postoperative problems in cardiac surgery. Better hemodynamic stability, less bleeding and shorter hospital and intensive care unit stays are all linked to it. Normothermic techniques may improve surgical results and patient recovery when used regularly.
